# D-Dimer: An Early Marker of Disseminated Intravascular Coagulation in Preeclampsia and Eclampsia

**DOI:** 10.7759/cureus.98809

**Published:** 2025-12-09

**Authors:** Rahul Sinha, Himanshu Joshi, Mitali Das, Rashmi Bhardwaj

**Affiliations:** 1 Pathology, Subdivision Hospital, Bermo, Bokaro Steel City, Jharkhand, IND; 2 Pathology, Uttar Pradesh University of Medical Sciences, Etawah, IND; 3 Pathology, All India Institute of Medical Sciences, Patna, Patna, IND; 4 Anatomy, Shri Siddhi Vinayak Medical College and Hospital, Sambhal, IND

**Keywords:** d-dimer, disseminated intravascular coagulation, parity, pih, preeclampsia, pregnancy-induced hypertension, thrombocytopenia

## Abstract

Introduction

Preeclampsia, a disease of unknown etiology, affects multiple organ systems and carries a grave prognosis for both the mother and fetus. Any hypertension and proteinuria developing after 20 weeks of gestation in a previously normal patient warrants investigation for this disease. Coagulation studies may be deranged in preeclampsia and eclampsia due to their association with disseminated intravascular coagulation (DIC). In DIC, the underlying basic pathology is endothelial dysfunction and intense vasospasm; it can affect any organ but most commonly affects the brain, kidneys, uterus, and placenta.

Materials and method

This retrospective cross‑sectional study was conducted on a sample size of 432 subjects over a two-year period in a tertiary referral center. Two categories of patients were identified and selected: preeclampsia patients and the control group, which comprised other admitted pregnant patients age-matched with the study group. Patient data were entered into Excel and analyzed using SPSS Statistics Version 24. Continuous variables were represented as mean and standard deviation (SD), while categorical variables were represented as frequency and percentage. Statistical analysis was performed using a parametric test of ANOVA. Significance level (p-value) was 0.05.

Results

Among the patients, 180 (41.7%) patients were nullipara, while 252 (58.3%) were multipara. All patients showed hypertension, with 382 (88.4%) patients having blood pressure values between 140/90 and 160/110 mmHg and 50 (11.2%) having values above 160/110 mmHg.

Among the patients, 191 (44.2%) had grade 2 proteinuria, 79 (18.2%) had grade 3 proteinuria, and 18 (4.2%) had grade 4 proteinuria, as determined by the dipstick method. Additionally, 148 (34%) subjects had platelet count < 150,000/mm^3^; 31 (7.1%) subjects had severe thrombocytopenia; and 73 (16.8%) subjects had D-dimer < 0.5 µg/mL, 247 (63.4 %) between 0.5 and 4 µg/mL, and 85 (19.6%) ≥4 µg/mL. The observed decrease in platelet count and fibrinogen levels with progressively increasing D-dimer is clinically relevant and in alignment with the pathophysiology of consumptive coagulopathy. The observed trend in our study is in line with the expected behavior of coagulation markers during ongoing increasing fibrinolytic activity.

Severe disease presentation is associated with higher proteinuria levels, overt thrombocytopenia, higher fibrinogen levels (unless a patient has landed into consumptive coagulopathy case, which shows decreased fibrinogen levels), and highly elevated D-Dimer levels.

Conclusion

Preeclampsia increases D-dimer concentrations beyond 0.50 mg/L, which is considered the normal threshold for D-dimer levels, resulting in a false-positive D-dimer test. The D-dimer test performed as an initial diagnostic measure can preclude the need for imaging and, along with other laboratory tests and clinical signs and symptoms, may aid in diagnosis even in non-classical cases, thus reducing fetal mortality.

## Introduction

Preeclampsia, a disease of unknown etiology, affects multiple organ systems and carries a grave prognosis for both the mother and fetus. Any hypertension and proteinuria that develop beyond 20 weeks of gestation in a previously normal patient warrant investigation for this disease [1]. The incidence in primigravida is around 10%, and the incidence in multigravida is around 5% [2]. Pregnancies at risk of preeclampsia are associated with overall increased risk of adverse fetal, neonatal, and maternal outcomes, such as preterm birth, intrauterine growth restriction, perinatal death, acute renal and/or hepatic failure, antepartum hemorrhage, postpartum hemorrhage, and, in severe cases, maternal death [3]. In India, the incidence of preeclampsia is estimated to be around 8-10% in pregnant women [4].

Preeclampsia may be an abnormal placentation state and is usually associated with maternal coagulation defects of hypercoagulability and fibrinolysis. The underlying disease process manifests as endothelial dysfunction and intense vasospasm, affecting multiple organ systems, particularly the uterus, kidney, placenta, and brain. Endothelial dysfunction results from oxidative stress and various inflammatory mediators. The underlying defect of vasospasm results from an imbalance between vasodilators such as prostaglandin I2 and nitric oxide and vasoconstrictors such as angiotensin-II, thromboxane A2 (TXA2), and endothelin-1 [5]. Additionally, altered levels of various biomolecules such as platelets, fibrinogen, antithrombin-III, and plasminogen are noted. The deranged physiology results in the formation of widespread microthrombi affecting placenta and various other organ systems, resulting in specific pathological changes.

The various laboratory parameters in preeclampsia are as follows: platelet levels are reduced, while certain markers such as D-dimer and thrombin levels are elevated.

The D-dimer molecule, a fibrin degradation product, is a small protein fragment that appears in the blood after a blood clot is degraded through fibrinolysis. D-dimer gets its name because it contains two D-fragments of the fibrin protein joined by a cross-link [6]. D-dimer levels are slightly elevated in normal pregnancy, but they are highly elevated in conditions of preclampsia and eclampsia, requiring urgent and prompt intervention.

The management and prognosis of the clinical condition of preeclampsia depend on the period of gestation, severity of disease, and timing of intervention. Timely diagnosis and intervention are necessary to achieve a favorable pregnancy outcome, which otherwise may carry a grave prognosis.

Performing the D-dimer test initially can exclude the need for imaging, and hence it is recommended as an initial diagnostic modality [6-9], demonstrating the importance of this study. Hence, the present study was undertaken to assess the correlation between D-dimer levels and markers of disease severity (platelets, proteinuria, fibrinogen) and to evaluate the potential of D-dimer use as an early marker for DIC in preeclampsia.

## Materials and methods

The present retrospective cross‑sectional study was carried out on 432 subjects at a tertiary care referral center over a two-year period. Ethical clearance for the study was obtained from the Institutional Ethics Committee of the institute (Ref. No. 120/2019). Inclusion criteria included all the admitted patients in the Department of Obstetrics and Gynecology during the study period showing clinical features of preeclampsia that warranted further laboratory investigations for proper management and treatment. Exclusion criteria included patients not willing to participate in the study, those with clotted samples, and those with incomplete clinical history.

The clinical records of the cases were obtained from the electronic patient database, and relevant medical histories, including blood pressure (BP), were obtained from case records of the Department of Obstetrics and Gynecology. Complete hemograms were obtained by running samples collected in EDTA vials on a Pentra XLR automated hematology analyzer (five-part hematology analyzer machine) (HORIBA ABX SAS, Montpellier Cedex 4, France). Subsequently, D-dimer assay was performed. Samples were collected in 3.2% sodium citrate containers. D-dimer assay was done in an ACL TOP 300 CTS fully automated coagulometer machine (Instrumentation Laboratory, Bedford, MA, USA). In the laboratory setting, the maximum value of D-dimer given by the automated coagulometer is 4 µg/mL. Above that value, the machine reports it as D-dimer > 4 µg/mL. Urine analysis was done using the dipstick method, using LabStrip U11 Plus Urine Analysis Dipstick Strips (77 Elektronika Kft., Budapest, Hungary), following instructions for use to detect proteinuria.

In the present study, two categories were identified: preeclampsia patients and the control group (which included other age-matched admitted pregnant subjects). Data entered into an Excel spreadsheet (Microsoft® Corp., Redmond, WA) were analyzed and evaluated using Statistical Package for the Social Sciences(SPSS) version 24 (IBM Corp., Armonk, NY). Normality of the continuous variables of our study were evaluated using the Kolmogorov-Smirnov test, along with visual inspection of histograms. The variables were found to be normally distributed and hence were summarized using mean ± standard deviation. The comparison was subsequently performed using a parametric test of ANOVA. The level of significance (p value) was considered as 0.05.

## Results

The study participants’ ages ranged from 18 years to 43 years, with a mean age of 26.5 years ± 5.02 years. Among the patients, 180 (41.7%) patients were nullipara, while 252 (58.3%) were multipara. Although some studies have suggested a trend toward higher coagulation markers in multiparous women, the data are not consistent across all populations. All patients showed hypertension, 50 (11.2%) patients had BP values above 160/110 mm Hg, and 382 (88.4%) had BP values between 140/90 and 160/110 mm Hg. The majority of the preeclamptic subjects, 191 (44.2%), presented with grade 2 proteinuria levels, 79 (18.2%) had grade 3 proteinuria, and 18 (4.2%) had grade 4 proteinuria by dipstick method. Among the subjects, 85 (19.6%) had highly elevated D-dimer levels (≥4 µg/mL), which is correlated with severe disease. Severe disease is also reflected in overt thrombocytopenia (i.e., a platelet count < 100,000/mL) and significantly increased fibrinogen levels. Those with very severe disease show decreased fibrinogen levels attributable to the process of DIC or consumptive coagulopathy. The observed decrease in platelet count and fibrinogen levels with progressively increasing D-dimer is clinically relevant and in alignment with the pathophysiology of consumptive coagulopathy. The observed trend in our study is in line with the expected behavior of coagulation markers during ongoing increasing fibrinolytic activity.

Regarding laboratory parameters, 73 (16.8%) subjects were noted to have normal D-dimer levels below 0.5 µg/mL, 247 (63.4 %) had elevated D-dimer levels between 0.5 and 3.9 µg/mL, and 85 (19.6 %) had highly elevated D-dimer levels greater than or equal to 4 µg/mL (Table 1).

**Table 1:** D-dimer level in patients.

D-dimer level (µg/mL)	No. of patients	Percentage (%)
<0.5 µg/mL	73	16.8
0.5-3.9 µg/mL	247	63.4
≥4 µg/mL	85	19.6

Of the 432 subjects, 148 had platelet levels less than 150,000/mm3. Of those 148 patients with low platelet levels, 58 had platelet levels between 100,000/mm3 and 150,000 (mild thrombocytopenia), 59 patients had platelet levels between 50,000/mm3 and 100,000/mm3 (moderate thrombocytopenia), and 31 patients had platelets levels above 50,000/mm3 (severe thrombocytopenia) (Table 2).

**Table 2:** Patients with platelet counts <150,000/mm3

Platelets level	No. of patients	Percentage (%)
100-150x1,000/mm^3^	58	13.4
100-50x1,000/mm^3^	59	13.6
<50,000/mm^3^	31	7.1

The majority of the cases, 191 (44.2%), had grade 2 proteinuria, 79 (18.2%) had grade 3 proteinuria, and a small number of preeclamptic patients, 18 (4.2%), had grade 4 proteinuria, as determined by the dipstick method. Higher levels of proteinuria were associated with severe disease presentation (Table 3). Accepting ≥2 + dipstick proteinuria may improve overall diagnostic accuracy for preeclampsia; however, in those cases, it is associated with a higher false-negative rate.

**Table 3:** Proteinuria by the dipstick method in patients of preeclampsia in the present study.

Urine dipstick	Number (N=432)	Percentage (%)
Nil/trace	16	3.7 %
1+ (≤30 mg/dL)	128	29.6 %
2+ (30-100 mg/dL)	191	44.2 %
3+ (100-300 mg/dL)	79	18.2 %
4+ (≥2,000 mg/dL)	18	4.2 %

In our study, 180 (41.7%) pregnant females were nulliparous, whereas 252 (58.3%) were multiparous (Table 4). Preeclampsia is said to affect nulliparous patients more than multiparous patients. However, the present study shows a higher incidence of preeclampsia in multiparous patients. This is attributed to the higher number of cases being referred for management to our tertiary care hospital.

**Table 4:** Parity distribution among patients.

Parity	Number (n)	Percentage (%)
Nullipara	180	41.7 %
Multipara	252	58.3 %
Total	432	100 %

Table 5 shows the minimum and maximum values of individual parameters with their mean and SD.

**Table 5:** Group descriptive analysis.

Test variables	Min. value	Max. value	Mean	Std deviation
Age (years)	18	43	26.5	5
D-dimer(µ/mL)	0.1	4	1.5	1.3
Serum fibrinogen (g/L)	0.7	6.7	4.1	1.2
Platelets (x1,000/mm^3^)	10	596	191	101.9

## Discussion

Hedengran et al. found that even normal pregnancies are associated with a gradual increase in D-dimer levels throughout pregnancy [10]. Considering the above findings and consistent with previously published studies, the current study also suggests that in pregnancy, the diagnostic threshold for D-dimer needs to be interpreted with caution and in relation to gestational age when considering the diagnosis of pregnancy-induced hypertension or preeclampsia [11,12]. The diagnosis of preeclampsia is routinely done by BP measurements and the estimation of urinary protein concentration, although newer supplementary tests have been introduced that can aid in suspected cases of preeclampsia [13]. However, it is generally noted that for improving overall diagnostic performance, the cut-off value of proteinuria by the dipstick method should be ≥grade 2 proteinuria, or, better yet, alternative tests such as spot urine protein/creatinine ratio should be used to screen for hypertension during pregnancy. In this context, D-dimer has emerged as a useful indicator for diagnosing various thrombotic states because its high concentrations are positively associated with the development or presence of venous thromboembolism. Manolov et al. found that in the last trimester of pregnancy, the development of preeclampsia is associated with elevated D-dimer levels [14]. Similarly, positive findings are suggested by the work of Bao et al. on levels of D-dimer in preeclampsia. In their study, Bao et al. pointed out that elevated D-dimer values relate positively with adverse feto-maternal outcomes [15]. Moreover, the concentrations of D-dimer are significantly correlated with the severity of clinical features of gestational hypertension [16,17].

In the current study, when comparing with gestational age-matched controls, D-dimer levels of preeclamptic women were significantly higher - a finding also reported by Tacoosian et al. [18]. Similar results were also reported by Kucukgoz Gulec et al., who found that D-dimer levels were significantly higher in patients with severe preeclampsia than those with mild preeclampsia [19]. The weighted overall effect showed by our study suggests the usefulness of testing D-dimer plasma levels in diagnosing preeclampsia (i.e., plasma D-dimer levels can easily be used as a screening test for the hypercoagulable state in preeclamptic patients). Additionally, this test may also be useful for determining prognosis outcomes, which have preventive and therapeutic implications, in this clinical condition of preeclampsia [20,21].

## Conclusions

Preeclampsia increases D-dimer concentrations beyond 0.50 mg/L, which is considered the normal threshold for D-dimer levels, resulting in a false-positive D-dimer test. However, the potential practical utility of performing this D-dimer test initially is that it can exclude the need for imaging, and hence it is recommended as an initial diagnostic modality/test, justifying the need for and importance of this study. Performing D-dimer assay first along with other laboratory investigations and monitoring clinical signs and symptoms, even in nonclassical cases, may help in early diagnosis, thus reducing maternal and fetal mortality. The findings in our study highlight the need for additional studies and supplementary tests throughout pregnancy, including the establishment of a gestational age-appropriate cut-off of D-dimer levels, to elucidate the diagnostic and prognostic role of D-dimer in preeclampsia. Further multi-institutional level studies with still larger sample sizes are needed for determining clinically useful cut-off values of D-dimer so as to make it a more dependable test.

## References

[1] Vázquez-Rodríguez JG, Barboza-Alatorre DY (2018). [Maternal and perinatal outcomes of expectant treatment of severe preeclampsia]. Rev Med Inst Mex Seguro Soc.

[2] Adam SS, Key NS, Greenberg CS (2009). D-dimer antigen: current concepts and future prospects. Blood.

[3] Fesmire FM, Brown MD, Espinosa JA, Shih RD, Silvers SM, Wolf SJ, Decker WW (2011). Critical issues in the evaluation and management of adult patients presenting to the emergency department with suspected pulmonary embolism. Ann Emerg Med.

[4] Torbicki A, Perrier A, Konstantinides S (2008). Guidelines on the diagnosis and management of acute pulmonary embolism: the Task Force for the Diagnosis and Management of Acute Pulmonary Embolism of the European Society of Cardiology (ESC). Eur Heart J.

[5] Qaseem A, Snow V, Barry P (2007). Current diagnosis of venous thromboembolism in primary care: a clinical practice guideline from the American Academy of Family Physicians and the American College of Physicians. Ann Fam Med.

[6] Dutta DC (2015). DC Dutta’s Textbook of Obstetrics. 8th Edition. 8th Edition, Jaypee Brothers Medical Publisher’s Ltd., New Delhi, 369.

[7] National High Blood Pressure Education Program Working Group (1990). Report on High Blood Pressure in Pregnancy. Am J Obstet Gynecol.

[8] (2025). Preeclampsia and Eclampsia. https://www.nichd.nih.gov/health/topics/preeclampsia.

[9] Steegers EA, von Dadelszen P, Duvekot JJ, Pijnenborg R (2010). Pre-eclampsia. Lancet.

[10] Hedengran KK, Andersen MR, Stender S, Szecsi PB (2016). Large D-dimer fluctuation in normal pregnancy: a longitudinal cohort study of 4,117 samples from 714 healthy Danish women. Obstet Gynecol Int.

[11] Kawaguchi S, Yamada T, Takeda M, Nishida R, Yamada T, Morikawa M, Minakami H (2013). Changes in d-dimer levels in pregnant women according to gestational week. Pregnancy Hypertens.

[12] Bremme KA (2003). Haemostatic changes in pregnancy. Best Pract Res Clin Haematol.

[13] (2002). ACOG Practice Bulletin No. 33: Diagnosis and Management of Preeclampsia and Eclampsia. Obstet Gynecol.

[14] Manolov V, Marinov B, Masseva A, Vasilev V (2014). [Plasma D-dimer levels in preeclampsia]. Akush Ginekol (Sofiia).

[15] Bao W, Xingshun Qi, Hongyu Li, Hou F, Zhang X, Wang R, Guo X (2017). Correlation of D-dimer level with the inflammatory conditions: a retrospective study. AME Medical J.

[16] Kim SJ, Ahn HJ, Park JY (2017). The clinical significance of D-dimer concentrations in patients with gestational hypertensive disorders according to the severity. Obstet Gynecol Sci.

[17] Baboolall U, Zha Y, Gong X, Deng DR, Qiao F, Liu H (2019). Variations of plasma D-dimer level at various points of normal pregnancy and its trends in complicated pregnancies: a retrospective observational cohort study. Medicine (Baltimore).

[18] Tacoosian Z,  Haj Seyed Javadi E,  Farzam S (2007). Evaluation of correlation between pre-eclampsia with D-dimer. J Inflamm Res.

[19] Kucukgoz Gulec U, Tuncay Ozgunen F, Baris Guzel A, Buyukkurt S, Seydaoglu G, Ferhat Urunsak I, Cuneyt Evruke I (2012). An analysis of C-reactive protein, procalcitonin, and D-dimer in pre-eclamptic patients. Am J Reprod Immunol.

[20] Pinheiro Mde B, Junqueira DR, Coelho FF, Freitas LG, Carvalho MG, Gomes KB, Dusse LM (2012). D-dimer in preeclampsia: systematic review and meta-analysis. Clin Chim Acta.

[21] Rahman R, Begum K, Khondker L, Majumder NI, Nahar K, Sultana R, Siddika A (2015). Role of D-dimer in determining coagulability status in pre-eclamptic and normotensive pregnant women. Mymensingh Med J.

